# In Vitro Correction of Point Mutations in the *DYSF* Gene Using Prime Editing

**DOI:** 10.3390/ijms26125647

**Published:** 2025-06-12

**Authors:** Camille Bouchard, Joël Rousseau, Gabriel Lamothe, Marie Dubost, Laura Bastrenta, Sina Ramezani, Jacques P. Tremblay

**Affiliations:** 1 Département de Médecine Moléculaire, Université Laval, Québec, QC G1V 0A6, Canada; 2 Centre de Recherche du Centre Hospitalier Universitaire de Québec, Québec, QC G1E 6W2, Canada; 3 Polytech Angers, Université d’Angers, 49045 Angers, France; 4 Faculté de Pharmacie, Université Grenoble-Alpes, 38400 Saint-Martin-d’Hères, France

**Keywords:** CRISPR, dysferlin, dysferlinopathy, gene therapy, LGMD, Miyoshi Myopathy, prime editing, point mutation

## Abstract

Dysferlinopathy is caused by over 500 mutations in the gene encoding dysferlin, including close to 300 point mutations. One option to cure the disease is to use a gene therapy to correct these mutations at the root. Prime editing is a technique which can replace the mutated nucleotide with the wild-type nucleotide. In this article, prime editing is used to correct several point mutations in the DYSF gene responsible for dysferlinopathy. In vitro editing of HEK293T cells reaches up to 31%. Notably, editing was more efficient in myoblasts than in patient-derived fibroblasts. The prime editing technique was also used to create a new myoblast clone containing a patient mutation from a healthy myoblast cell line.

## 1. Introduction

There are 39 types of limb–girdle muscular dystrophies (LGMDs), each caused by one or more mutations in a different gene [1,2]. Dysferlinopathy is one type of LGMD and is caused by mutations in the DYSF gene (MIM#603009, GenBank NM_003494.2), encoding dysferlin [3,4]. This protein has seven C2 domains (C2A to C2G), which are involved in localizing the T-tubules, repairing the sarcolemma by fusing vesicles, and Ca^2+^ signaling [5,6]. Several types of mutations in the dysferlin gene are found in the patient population. Some patients have a partial deletion of an exon, others have an insertion that causes a change in the reading frame, and finally, many patients have a point mutation (i.e., insertion, deletion, or modification of a few nucleotides) [7]. Point mutations often result in the substitution of one amino acid for another or the formation of a premature stop codon.

The main symptoms of dysferlinopathy are a gradual loss of muscle strength in the proximal girdle muscles (whose clinical presentation is called LGMD 2B or R2), mainly in the gluteal muscles, the tensor fascia latae, and in the posterior section of the thigh (adductors, hamstring) [8]. This loss of strength first causes difficulty tiptoeing and then difficulty climbing stairs during a person’s teenage years or early twenties [9]. The other most common clinical presentation is that of Miyoshi’s muscular dystrophy, which first affects the distal muscles, such as the gastrocnemius and soleus [10,11]. After five to ten years following the onset of symptoms, the two presentations become similar and are grouped under the term dysferlinopathy, since the same mutation can cause both [12,13].

Since dysferlinopathy is a recessive disease, patients who exhibit symptoms will either have a homozygous mutation or a combination of heterozygous mutations. Several patients have point mutations that usually cause a premature stop codon, resulting in the absence of dysferlin in the patient [7]. There is currently no approved treatment targeting the symptoms for this disease, with most interventions seeking to reduce the signs and symptoms of the disease (painkillers, orthotics, walking aids, home adaptations) but failing to slow its progression [14,15]. Some of these therapies aim to reduce inflammation and oxidative stress in the muscles. As such, most of the current research seeking to treat this disease focuses on gene editing and delivering a functional protein.

Various approaches have been, and are continuing to be, developed to correct hereditary diseases such as dysferlinopathy. Since these diseases are often caused by the lack of a functional protein in the patient, some teams have attempted to deliver the missing protein, cells producing it [16], or the gene required to produce it. In 2003, a German study (NCT00527228) assessed the effects of deflazacort in patients with dysferlinopathy. This double-blind, placebo-controlled trial involved 25 genetically confirmed patients who received either deflazacort or the placebo over six months, following a one-year natural history phase. The results indicated that deflazacort did not improve muscle strength and was associated with a trend toward worsening strength, which reversed upon discontinuation. Additionally, patients experienced a broad spectrum of steroid-related side effects. This study concluded that deflazacort is not an effective therapy for dysferlinopathies and should not be used off-label, especially since these conditions can be misdiagnosed as polymyositis, for which steroids are typically prescribed [17]. In the United States, a Phase I clinical trial (NCT02710500) evaluated the intramuscular injection of rAAVrh.74.MHCK7.DYSF.DV into the extensor digitorum brevis muscle from March 2016 to July 2019. Preclinical studies in mice and non-human primates demonstrated restored dysferlin expression and improved muscle function, including membrane repair and diaphragm strength. However, the clinical trial results have not yet been published [18]. Another ongoing clinical trial (NCT05906251) is investigating the safety, efficacy, and tolerability of SRP-6004, a dual-vector AAV gene therapy administered intravenously to ambulatory patients with limb–girdle muscular dystrophy type 2B/R2 (LGMD2B/R2), a form of dysferlinopathy. This open-label, Phase I study began in May 2023 and is expected to be completed in August 2028. Alternatively, other approaches look to modify the patient’s existing gene to correct the pathogenic mutation(s) [3,19]. To accomplish this, technologies such as Transcription Activator-Like Effector Nuclease (TALEN) [20], Zinc Finger Nucleases (ZFNs) [21,22], Clustered Regularly Interspaced Short Palindromic Repeats (CRISPRs), base editors [23,24], and prime editors (PE) have been developed.

The most recent of these techniques is prime editing, a technology derived from CRISPR. At the heart of this technology is a Cas (CRISPR-associated) protein. Cas9, one amongst many different types of Cas proteins, has evolved in some prokaryotes as a defense against phages. Cas9 can be programmed using an RNA sequence to recognize phage DNA and cleave it before the phage takes over [25]. Prime editing uses a mutated Cas9 nickase (nCas9) fused with a reverse transcriptase (RT) to install new mutations in a DNA sequence. To do this, the Cas protein is programmed by a prime editor guide RNA (pegRNA) to recognize a DNA sequence and cut a single strand in the duplex. The ends of this cut can then be modified to rewrite the existing DNA sequence based on the template in the pegRNA [26]. There are several generations of PE (from PE1 to PE7) [27]. We used the traditional PE2 as well as the PE3 technique [26], which contains an additional nicking sgRNA (nsgRNA) to cut in the DNA strand that is not targeted by the epegRNA to favor the retention of the nucleotide modification in the strand modified by the epegRNA. They all include an nCas9 fused with an RT and a pegRNA. The pegRNA is composed of a constant part to give rise to the structure as well as a personalized part, including the protospacer, to allow the cut to happen precisely at the desired site, the Primer Binding Site (PBS), allowing for the open DNA strand to then bind where it will be easily accessible for the RT and a Reverse Transcriptase Template (RTT) to provide the new sequence to be written on the edited strand. The RTT usually contains the RNA sequence to be used as a template to either correct or insert a mutation, and can contain synonymous mutations to track in WT cells or to modify the PAM. Adding a synonymous mutation in the PAM also improves the efficiency, as shown by Mbakam et al. [28].

Recent work on prime editing confirms that it is possible to modify the DNA of cells in the human body at a level sufficient to treat several pathologies [29,30,31]. However, a method to effectively deliver the components of the prime editing system to diseased cells is the subject of ongoing work [32,33].

Since prime editing is able to correct a point mutation without modifying the nucleotides that precede or follow it, which is not the case with base editing, it could correct point mutations in the DYSF gene while reducing the probability of off-target mutations.

## 2. Results

### 2.1. Insertion of Synonymous Mutations Using Plasmids to Correct Patient Point Mutations in HEK293T Cells

**Figure 1:**
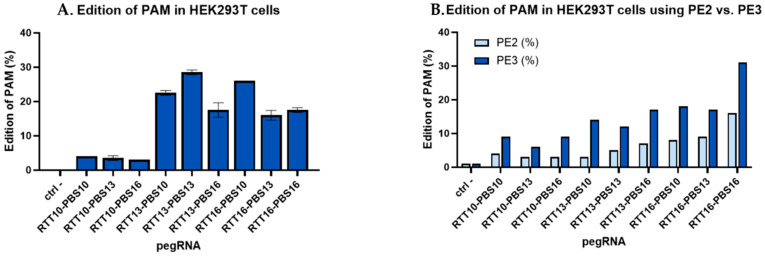
Percentage of editing in HEK293T by PE constructions made to correct the human *DYSF* E1833X and *DYSF* R1905X mutations. (**A**) Various pegRNA lengths designed to correct the *DYSF* E1833X mutation used in HEK293T cells: Different prime editing constructs made it possible to evaluate the optimal lengths of RTT and PBS to correct a given point mutation. For the E1833X mutation, this size was 13 nucleotides for both RTT and PBS. This construction made it possible to obtain 29% editing by transfection with Lipofectamine 2000 in HEK293T cells. (**B**) Comparing PE2 and PE3 using pegRNAs designed to correct the *DYSF* R1905X mutation used in HEK293T cells: Using an additional nicking single guide RNA (nsgRNA) to cut the DNA strand not modified by the epegRNA doubled the percentage of editing obtained, with epegRNA containing various RTT and PBS lengths. The figure illustrates PE2 (in light blue), using epegRNA to cut the DNA near the mutation, and PE3 constructs (in dark blue), using an additional nsgRNA, which are, on average, twice as effective at inserting a tracking mutation and allowed up to 31% of tracking mutations to be obtained in the HEK293T cells when the RTT contained 16 nucleotides and the PBS also contained 16 nucleotides. The experiment was repeated 3 times in 2 wells for each pegRNA construction.

Prior to receiving the fibroblasts from patients, PE plasmids encoding pegRNAs that could correct patient mutations were tested on HEK293T cells. While the HEK293T cells did not have the patient mutations, the editing efficiencies of these pegRNAs could still be determined by comparing the editing efficiency of a “tracking” mutation. Some, but not all, of the pegRNA created a synonymous mutation in the PAM to increase editing efficacy when correcting patient mutations. In the context of the HEK293T cells, this PAM mutation could be tracked to compare the effectiveness of the different RTT and PBS lengths. The results indicated that the optimal sizes of RTT and PBS varied for each patient mutation. For example, Figure 1A illustrates that the plasmid constructed to correct the DYSF E1833X mutation in the DYSF gene had an efficiency of 29% when the RTT and PBS were both 13 nucleotides long. For plasmids aimed at correcting the DYSF R1905X mutation, 31% editing was observed when both RTT and PBS were 16 nucleotides long (Figure 1B).

We used the PE3 technique [26], which contains an additional nicking sgRNA (nsgRNA) to cut the DNA strand that is not targeted by the epegRNA. This favored the retention of the nucleotide modification in the strand modified by the epegRNA [34]. Figure 1B shows that using PE3 instead of the PE2 technique doubled the percentage of edits in HEK293T for a synonymous mutation through the construction targeting the DYSF R1905X mutation.

### 2.2. Correction of Patient Point Mutations in Fibroblast Lines Derived from Patient Skin Biopsies

**Figure 2:**
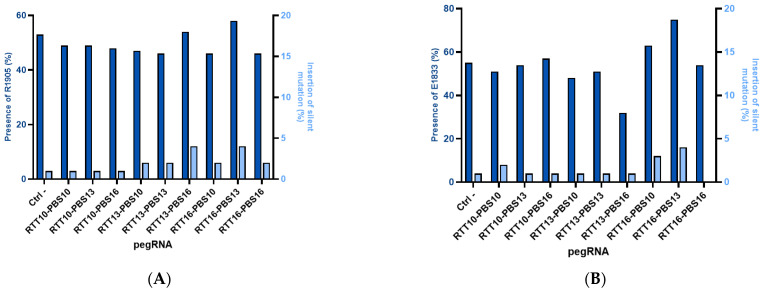
Editing percentage in patient-derived fibroblasts. (**A**) *DYSF* R1905X: Editing in patient-derived fibroblasts has a maximum of 4% for the synonymous mutation added in the PAM and 5% for the correction of the patient’s R1905X mutation. The negative control with a plasmid containing the GFP gene indicates that 53% of the DNA does not have a pathological mutation, and the highest result is 58% for the RTT16-PBS13 construct (corresponding to 5% editing). (**B**) E1833X: The presence of E1833 increases by 20% (Ctrl = 55% E1833 vs. 75% E1833 for RTT16-PBS13), but the synonymous mutation of the PAM remains implanted at 0–4%.

Among the plasmids previously used in HEK293T, the most efficient were chosen to be electroporated into fibroblasts derived from patient biopsies. It was noted that prime editing in fibroblasts was less efficient when compared to 31% prime editing in HEK293T cells. Up to a 20% correction of the patient mutation was observed in these fibroblasts (E1833X RTT16-PBS13), and a maximum 4% insertion of the synonymous mutation from the same RTT was obtained (Figure 2). The negative control in this figure shows 1% synonymous mutations as a background from Sanger sequencing.

The same trend has been seen in other projects in our laboratory when attempting to modify patient fibroblasts with a gene normally expressed in the muscles [28,35].

### 2.3. Gene Correction in Myoblast-like Cells Derived from Patient Fibroblasts

**Figure 3:**
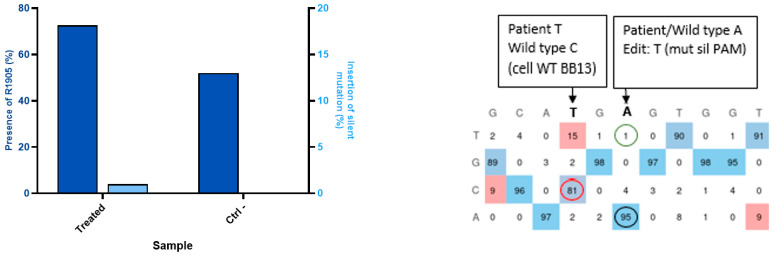
Editing in patient-derived fibroblasts expressing MyoD. The patient-derived fibroblasts with the R1905X mutation were cultured with doxycycline to induce the expression of the lentivirus-inserted *MyoD* gene, and the patient fibroblasts were heterozygous (52% of the *DYSF* genes were wild-type on average when sequenced). The DNA after prime editing treatment was found to contain the wild-type *DYSF* gene in between 61% and 81% (red circle) (average 71%) of the cells. Thus, there was a 20% increase in the wild type genes. This means that 40% of the disease alleles had been corrected. The synonymous PAM mutation T is introduced at 1% (green circle) and the WT A (black circle) is present at 95%. The EditR Software colors the desired nucleotides from the sequence in blue and the undesired (background or spontaneous mutations) or voluntarily modified nucleotides in red as they differ from the given wild-type sequence. The experiment was repeated 3 times in 2 wells for each pegRNA construction.

Heterozygous patient-derived fibroblasts were induced to express the MyoD gene to render them myoblast-like cells. In these cells, prime editing was used to correct up to 31% of pathogenic mutations (from the pathogenic T cells at 50% to C WT cells at 81% in Figure 3). However, the synonymous mutation in the same sequence is present with only 1% of T cells (Figure 3).

### 2.4. Avoiding Off-Target Mutation

**Figure 4:**
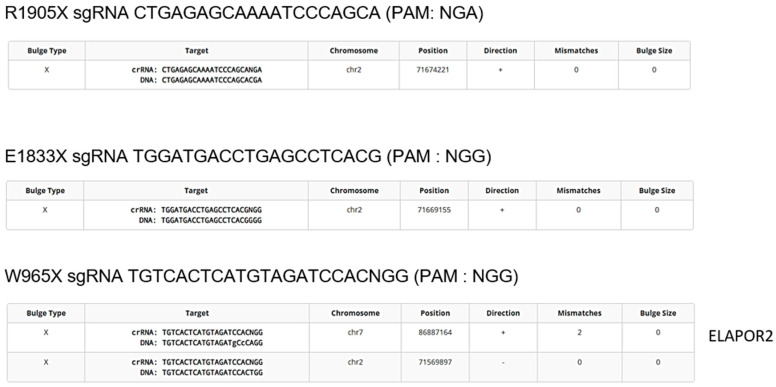
Detection of potential off-target mutations. The online software Cas-OFFinder identifies genome sequences similar to those targeted by the spacer of the epegRNA. The constructs to correct the *DYSF* R1905X, E1833X and Q1010X mutations theoretically do not target any other sequence in the genome. However, the protospacer sequence near the W965X mutation is similar to a sequence in the *ELAPOR2* gene (2 mismatches in the 20-nucleotide protospacer sequence) encoding the synthesis of a ubiquitous protein.

An effort was also made in trying to reduce off-target editing in the genome. Therefore, the protospacer sequences were compared to the human genome to evaluate the similarity between them. If a protospacer sequence and a PAM are present somewhere else, the DNA may also be cut and edited there too. Cas-OFFinder (CRISPR RGEN Tools (rgenome.net), accessed on 17 April 2025) suggested that the protospacer sequences used to correct *DYSF* R1905X and E1833X did not have similarity to other parts of the human genome, yet the protospacer used to correct and insert the *DYSF* W965X mutation was similar to a sequence in the *ELAPOR2* gene with two mismatches out of twenty nucleotides (Figure 4). The *ELAPOR2* gene codes for endosome/lysosome-associated apoptosis and autophagy regulator family member 2 protein. This protein regulates the bone morphogenetic protein (BMP) signaling pathway [36] and may be involved in epidermal differentiation (https://www.proteinatlas.org/ENSG00000164659-KIAA1324L [consulted on 27 May 2025]).

**Figure 5:**
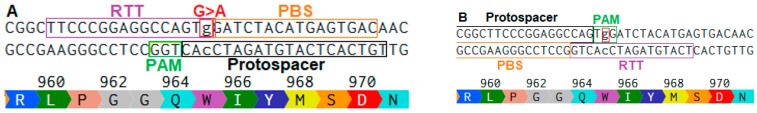
Two different protospacers may be used to insert the *DYSF* W965X mutation by prime editing. The position of the W965X (i.e., TGG (W)>TAG(Stop)) mutation in the *DYSF* gene is identified with a red square in bot (**A**,**B**). This figure illustrates that to insert this mutation, there are two possible PAMs and thus two possible protospacer sequences. Indeed, there is a TGG PAM in the bottom strand in Figure (**A**) and a TGG PAM in the top strand in Figure (**B**). In both cases, the protospacer sequences are composed of the 20 nucleotides located 5′ to the PAM. The break is performed at 3 nucleotides from the PAM in the 5′ direction. The cut site is the boundary between the RTT and the PBS. Their lengths are variable and specific to each sequence. However, only the PAM in Figure (**A**) can be used to correct the patient mutation, since the PAM in Figure (**B**) uses the wild type. Capital letters and their colour indicate the amino acid associated with the DNA sequence.

Sometimes, there are no PAM sequences available near a mutation of interest. Alternatively, there are for other point mutations several different nearby PAM sequences. This is the case for installing the W965X mutation in the DYSF gene. One of the epegRNAs targeting that mutation used a twenty-nucleotide protospacer sequence, which had only two mismatches with a sequence present in the ELAPOR2 gene, which could result in an off-target mutation in the ELAPOR2 gene. Therefore, since another PAM was possible, another epegRNA targeting a protospacer sequence in the opposite DYSF strand could be used for inserting the patient mutation in a healthy cell line to remedy to this problem (Figure 5). However, only the PAM in Figure 5A can be used to correct the patient mutation, since the PAM in Figure 5B uses the wild type.

### 2.5. Tracking Synonymous Mutations Using epegRNA to Insert a Synonymous PAM Mutation in Healthy Myoblasts

**Figure 6:**
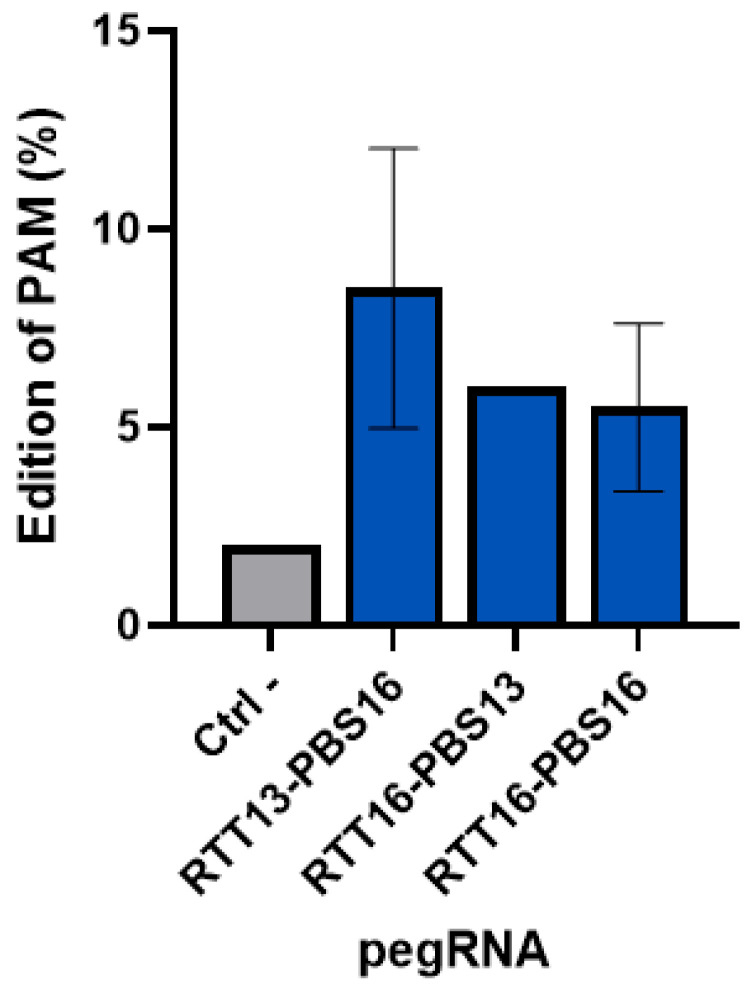
Percentage of insertion of the PAM synonymous mutation in healthy human myoblasts. A synonymous mutation was inserted in healthy human myoblasts in the PAM sequence used by 3 epegRNAs (with different RTT and PBS lengths). An average of 8.5% editing was obtained with the epegRNA containing an RTT with 13 nucleotides and a PBS with 16 nucleotides. The maximum insertion of the mutation was 11%. Each pegRNA was tested in two wells and the experiment was repeated a second time.

Following the electroporation of plasmids coding for the components of the prime editing technology (i.e., the prime editor, an epegRNA, and an additional nsgRNA) in healthy myoblasts, an average editing of about 8.5% was obtained with a maximum of 11% for one of the epegRNAs (Figure 6). The epegRNAs designed to correct the R1905X mutation in human cells were used since they also introduced a synonymous mutation in the PAM. This editing was tracked.

### 2.6. Creation of a Patient Mutation Cell Line (W965X) from Healthy Myoblasts Modified by Prime Editing

**Figure 7:**
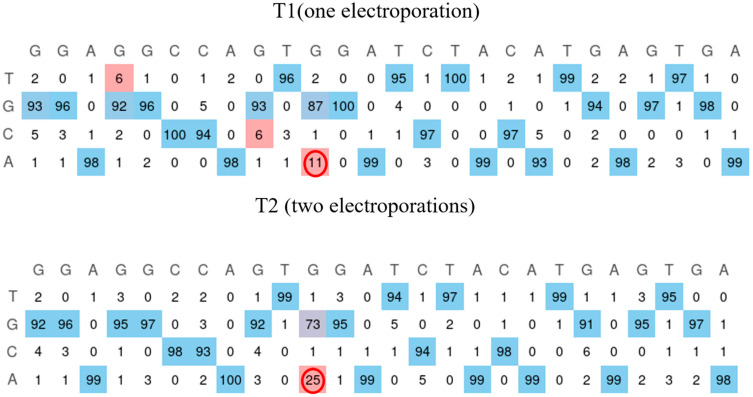
Insertion of the W965X mutation in healthy myoblasts. The human myoblasts cell line containing the W965X patient mutation is created by incorporating the mutation through electroporation of the plasmid of the prime editing components. The first electroporation makes it possible to obtain the mutation in 11% of the cells. The second treatment allows this figure to reach 25% (red circle). The EditR Software colors the desired nucleotides from the sequence in blue and the undesired (background or spontaneous mutations) or voluntarily modified nucleotides in red as they differ from the given wild-type sequence.

The W965X mutation is in the Jain Foundation’s patient registry mutation list, but no cell lines were available with this mutation. A cell line from healthy myoblasts was created through the repeated electroporation of the plasmid coding for the prime editing components to insert the pathogenic mutation. The mutation was present in 11% and 25% of cells following the first and second electroporation treatments (Figure 7). The cells were then grown individually to make a clone. The sequencing of the wells indicates the presence of the mutation in 20 of the 96 wells.

## 3. Discussion

Before receiving patient-derived fibroblasts, a synonymous mutation was inserted into HEK293T cells in the PAM used by epegRNAs that could correct the DYSF patient mutations. This synonymous mutation was inserted by the plasmids, which would eventually be used to correct various patient point mutations. This synonymous mutation allowed us to monitor the efficacy of various epegRNAs while waiting for the patient-derived fibroblasts. Since the patient mutations are not present in the HEK293T cell line, the synonymous mutation in the PAM was tracked to evaluate the efficiency of prime editing. The RTT13/PBS13 epegRNA (i.e., containing an RTT with 13 nucleotides and a PBS also with 13 nucleotides) targeting the DYSF E1833X mutation edited the PAM in up to 29% of the DYSF genes in HEK293T cells. The RTT16/PBS16 epegRNA designed to correct the DYSF R1905X mutation edited up to 31% of the PAM when using the PE3 method using an additional nsgRNA. Mutating the PAM also improves the PE efficiency [28], as well as adding an nsgRNA to cut further on the opposite strand [26]. Anzalone et al. also obtained between 20 and 55% editing using the PE3 method. The percentage of editing depended on the position of the nsgRNA and the position of the mutation [26]. We also confirmed in Figure 1B that the PE3 method doubled the prime editing efficiency compared to the PE2 method. Our results are thus similar to the 1.5- to 4.2-fold improvement observed by Anzalone et al. when comparing PE2 to PE3 [26].

Using the PE3 method to correct point mutations in fibroblasts derived from patient skin biopsies did not show clear and significant editing for all cell lines. A trend was observed wherein some RTT-PBS combinations seemed to correct the patient mutation by up to 20% while only installing the nearby synonymous PAM mutation in 4%. Mbakam et al. observed the opposite effect when attempting to correct a mutation in the DMD gene, correcting up to 13% of the pathogenic mutations and installing the PAM synonymous mutation in up to 36% of alleles with the PE3 method. We noticed that concurrent mutations created by the same RTT can be installed with different editing efficiencies, whether one is a patient mutation to correct or a synonymous mutation in the PAM or outside the PAM [37]. The three-dimensional structure of the RTT interacting with the DNA could explain why some nucleotides are edited more efficiently than their surrounding nucleotides [38].

When correcting MyoD-expressing patient-derived fibroblast cell lines to mimic myoblasts, the same trend was noticed, with an apparent mutation correction of 31%, but the insertion of the PAM synonymous mutation was observed in only 1% when using PE3. This leads us to question why inserting a synonymous mutation in the PAM increases the efficiency of PE yet leads to a lower insertion of the said mutation in the DNA. The 3D structure of the pegRNA might be responsible for this and needs to be studied in more detail [38]. Since we compared the correction of the same mutation (e.g., R1905X) using the same pegRNA in different cell lines (HEK293T, patient-derived fibroblasts with or without MyoD, and myoblasts), we hypothesized that more DNA was modified in HEK293T cells since they do not undergo efficient mismatch repair [39]. We also noticed in several experiments that more DNA modification was observed in myoblasts than fibroblasts for mutations causing muscle-wasting diseases [28,35]. Our hypothesis is that since these genes are expressed in muscles and not in skin, their DNA might not be accessible in fibroblasts (but may be held by histones).

Tracking synonymous PAM mutations using PE3 plasmids to model the correction of patient point mutations in healthy myoblasts resulted in up to 11% editing. Further tests will evaluate what percentage of correction of a mutation in the DYSF gene is sufficient to restore enough protein expression to ensure muscle repair.

Also, inserting a patient mutation in healthy myoblasts can not only be used to verify the effect of gene therapy but could also be used to study several parameters as a disease model cell line.

## 4. Materials and Methods

### 4.1. Selecting Patient Mutations

**Figure 8:**
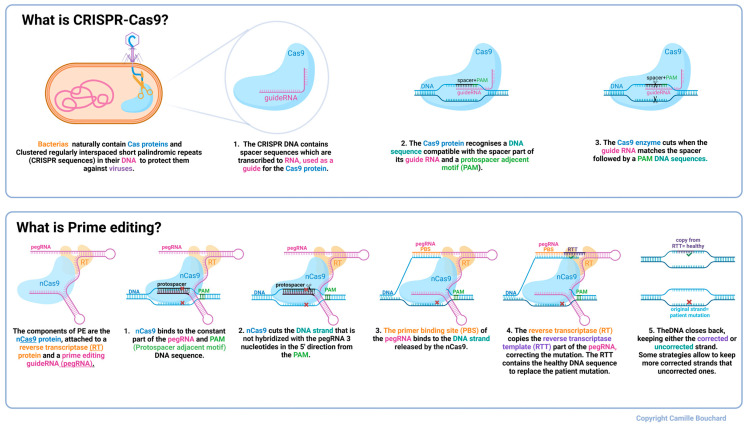
Description of CRISPR-Cas9 and prime editing. Colours in the text identify structures of the same colour in the figure above. The red X represents the patient mutation.

In this study, the Jain Foundation’s Patient Mutation Registry was consulted to select patient point mutations deemed amenable to prime editing (explained in Figure 8) because of the presence of an NGG protospacer adjacent motif (PAM) close to the mutation. Indeed, Cas9 nickase recognizes a PAM to cut 3 nucleotides in the 5′ direction [26]. The PAM sequence of the SpCas9n is NGG; however, new genetically modified SpCas9 enzymes, such as the one with a VQR mutation, also make it possible to recognize other PAM sequences such as NGAG [40]. There are also some Cas9 enzymes extracted from other bacteria that recognize a different PAM [41,42,43]. Therefore, the DYSF W965X, E1833X, and R1905X mutations were selected to be corrected in vitro since they respect the previously stated conditions. They are all premature termination codon (PTC) mutations, but all types of point mutations were considered for this study. W965X patients in the registry were homozygous, and E1833X and R1905X were compound heterozygous. Supplementary Table S2 states their mutations.

### 4.2. Plasmid Construction

**Figure 9:**
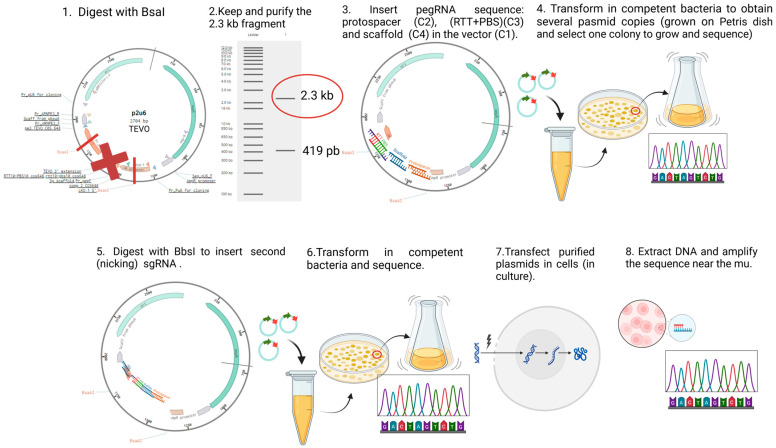
Construction and use of plasmids for prime editing. For the fabrication of prime editing plasmids, we used the protocol initially developed by Dr. David R. Liu’s team [26]. The first step was to digest the P2U6 plasmid (made from the pU6-tevopreq1-GG-acceptor (Addgene #174038), to which our laboratory added a second U6 promoter to insert the nsgRNA sequence) with the BsaI enzyme, removing the part with a red X and to insert the PBS and the RTT sequences between the sticky ends generated by the digestion. A transformation allowed for the isolation of a colony with the right sequence. The resulting plasmid (illustrated by a circle with arrows) was digested again with BbsI to add the sequence of a nicking single guide RNA (nsgRNA) to cut the DNA strand not modified by the epegRNA. A second transformation allowed for the selection of a bacterial colony containing the plasmid with the desired sequence. These plasmids were then used to treat different types of cells co-transfected with PE2-CMV (Addgene # 132775) by electroporation (Neonfection) or lipofection (Lipofectamine 2000, Thermo Fisher, Waltham, MA, USA). The DNA from the treated cells was extracted after 72 h, the DNA region near the targeted mutation (250–300 nucleotides) was PCR-amplified, and the amplicons were Sanger-sequenced and the sequence was analyzed with EditR software online (http://baseeditr.com/, last consulted on 7 June 2025). The colours used in the graph indicate the sequence nucleotides.

We constructed plasmids containing the sequence to express the prime editing components, i.e., the prime editor (a Cas9 nickase fused with an engineered reverse transcriptase) and an epegRNA, to correct the patient mutations selected above. For each mutation, we created nine plasmids in which the components of the epegRNA (i.e., the PBS and the RTT) varied in length (Supplementary Table S1). Figure 9 illustrates the steps followed to construct the prime editing plasmids, following the protocol described by Anzalone et al. [26]. Golden Gate assembly was used to insert the designed oligonucleotides (synthetized by Integrated DNA Technologies, Coralville, IA, USA). Supplementary Figure S1 illustrates the prime editing constructions for each patient mutation, here represented with a red X in Figure 8.

### 4.3. In Vitro Prime Editing of HEK293T

The Prime editing plasmids were first transfected with Lipofectamine 2000 in HEK293T cells. HEK293T cells were cultured in Dulbecco’s Modified Eagle Medium (DMEM) (Wisent Inc., Saint-Jean-Baptiste, QC, Canada) with 10% Fetal Bovine Serum (FBS) (Fisher Scientific Inc., Pittsburgh, PA, USA) and 1% Penicillin + streptomycin 100x (Wisent Inc.). Then, 72 h after transfection, the cells were washed in phosphate-buffered saline (PBS) 1X (Wisent Inc.) and centrifuged. To extract the DNA, the cell pellet was resuspended in 100 µL of direct PCR (Viagen Inc., Cedar Park, TX, USA) and 1 µL of proteinase K (20 ng/mL), incubated at 56 °C for 60 min, and inactivated at 85 °C for 45 min. DNA sequences (150 to 300 nucleotides) around the mutation were PCR-amplified to determine the frequency of the editing by Sanger sequencing and analysis with EditR software (EditR: Edit Deconvolution by Inference of Traces in R).

### 4.4. Culture of Patient Fibroblasts

Patient-derived immortalized fibroblast lines provided by the Jain Foundation were cultured in myoblast culture medium: 252 mL of Dulbecco’s Modified Eagle Medium (DMEM), Wisent (Saint-Jean-Baptiste, QC, Canada), 80 mL of Fetal Bovine Serum (FBS) (Fisher Scientific Inc., Pittsburgh, PA, USA), 64 mL of M199, pH 7.2 (Wisent Inc.), 100 µL of Fetuin from Fetal Bovine Serum (Sigma-Aldrich Inc., Saint-Louis, MO, USA), 20 µL of Gibco Human Epidermal Growth Factor (hEGF) (Fisher Scientific Inc.), 2 µL of Gibco Basic fibroblast growth factor (BFGF) Recombinant Human Protein (Fisher Scientific Inc.), 519 µL of Insulin, 100 IU/mL of Humulin R (Lilly Inc., Indianapolis, IN, USA), 2 mL of Dexamethasone (Sigma-Aldrich Inc.), and 4 mL of Penicillin streptomycin 100x (Wisent Inc.).

### 4.5. Plasmid Delivery and Sanger Sequencing

Plasmids encoding the prime editing components were electroporated into fibroblast cells using the 1100 volt, 20 ms, and 2 pulse setting (well 13) of the Neon MPK5000R (Invitrogen Fisher Scientific Inc., Waltham, MA, USA). Cellular DNA was extracted 72 h after the electroporation and the mutated sequence was PCR-amplified. Amplicons were sequenced using Sanger sequencing and the frequency of editing was determined using EditR (EditR: Edit Deconvolution by Inference of Traces in R).

### 4.6. Generation of Myoblast-like Cell Lines

Patient-derived fibroblasts were treated with two lentiviruses by Dr Monkol Lek’s team (https://www.jain-foundation.org/past-projects/the-use-of-crispr-as-a-potential-therapeutic-for-dysferlinopathy/, accessed on 22 January 2025). The first lentiviral plasmid (pLV-hTERT-IRES-hygro Addgene #85140) immortalized the fibroblasts and conferred upon them a resistance to hygromycine. The second lentiviral plasmid (LV-TRE-VP64 human MyoD-T2A-dsRedExpress2 Addgene #60629) enabled the expression of the MyoD gene to differentiate fibroblasts into myoblasts and induce puromycine resistance. Their expression was induced by adding 3 μg/mL of doxycycline to the medium. This was carried out to determine whether it was easier to modify the DYSF gene in the ‘myoblast-like’ cells as compared to fibroblasts. Prime editing in the myoblast-like cell lines followed the same protocol as that detailed for the fibroblasts.

### 4.7. Creation of a Patient Mutation Cell Line (W965X) from Healthy Myoblasts Modified by Prime Editing

Since patient-derived cell lines are not available for all mutations in the Jain Foundation’s registry, our team created a cell line from immortalized healthy myoblasts. PE plasmids were electroporated into healthy myoblasts in order to obtain a homozygous clone with the DYSF W965X mutation. Manual cloning was then performed to obtain a cell line with the patient mutation. In addition, the spacer sequence was verified with cas-OFFinder (CRISPR RGEN Tools (rgenome.net)) to evaluate its similarity to another human genome sequence, which could cause off-target mutations in other genes.

## 5. Conclusions

This article is the first step in a wider study. It was carried out to ascertain certain trends, and further steps will be performed in the future, such as verifying the occurrence of off-target mutations in the whole genome and deciphering at what percentage of correction the dysferlin function is restored. The in vivo delivery of PE packed in dual-AAV is also planned, as well as in other vectors such as extracellular vesicles. The whole scientific community is also working on finding efficient and safe delivery methods for cell and gene therapies in the hope to treat hereditary diseases such as dysferlinopathy.

## Data Availability

The original contributions presented in this study are included in the article/Supplementary Materials. Further inquiries can be directed to the corresponding authors.

## Supplementary Materials

The supporting information can be downloaded at: https://www.mdpi.com/article/10.3390/ijms26125647/s1.
