# Supplementary material for: In Vitro Correction of Point Mutations in the DYSF Gene Using Prime Editing
Source: Int J Mol Sci. 2025 Jun 12;26(12):5647. doi: 10.3390/ijms26125647 (PMC12193300; doi:10.3390/ijms26125647)
Supplement: Supplementary file 1 [file ijms-26-05647-s001.zip › ijms-3642460-supplementary.pdf]

W965X

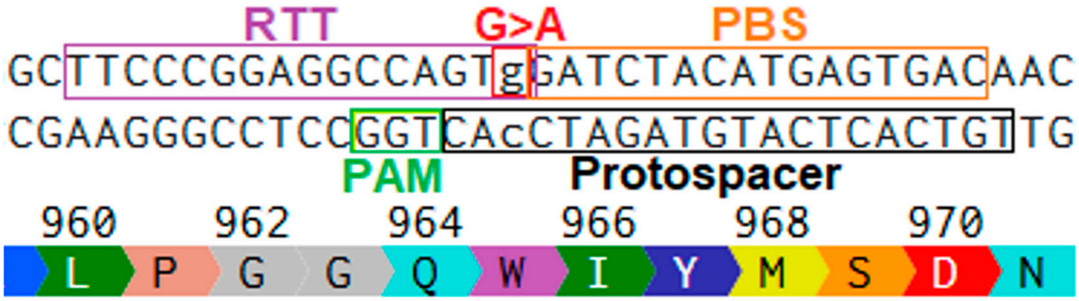

E1833X

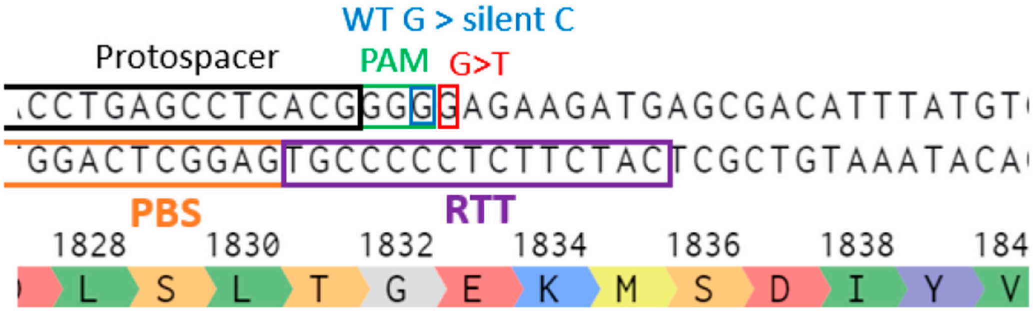

R1905X

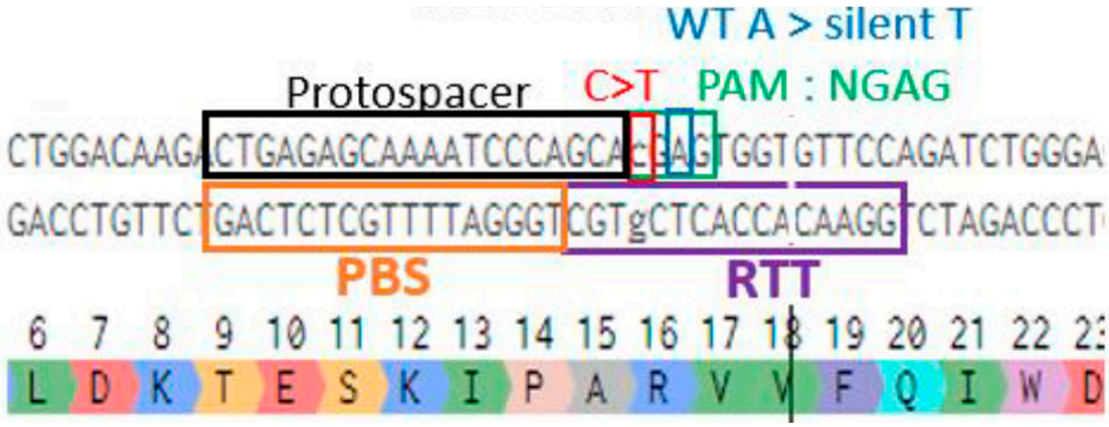

Supplementary Figure S1: Prime editing constructions to correct patient point mutations in the *DYSF* gene. The NGG or NGAG PAM was first identified to cut 3 nucleotides in the 5' direction within the 20 nt protospacer sequence. The PBS and RTT sequences were then identified. The RTT sequence was designed to correct the mutation and when possible, to insert a silent mutation in the PAM.

Supplementary Table S1: oligonucleotide sequences in PE plasmids.

**Lavoie W965X (PAM: NGG)En attente de la biopsie, ne pas commmander**	
**Nom**	**5' -> 3'**
Comp 2 (spacer):	
Comp2 Up Lav	caccgTGTCACTCATGTAGATCTACgtttt
Comp2 Lo Lav	ctctaaaacTGTCACTCATGTAGATCTACc
Comp 3 (RTT + PBS) :	
RTT10-PBS10 Up Lav	gtgcGAGGgCAGTGGATCTACATG
RTT10-PBS10 Lo Lav	cgcgCATGTAGATCCACTGcCCTC
RTT10-PBS13 Up Lav	gtgcGAGGgCAGTGGATCTACATGAGT
RTT10-PBS13 Lo Lav	cgcgACTCATGTAGATCCACTGcCCTC
RTT10-PBS16 Up Lav	gtgcGAGGgCAGTGGATCTACATGAGTGAC
RTT10-PBS16 Lo Lav	cgcgGTCACTCATGTAGATCCACTGcCCTC
RTT13-PBS10 Up Lav	gtgcCCGGAGGgCAGTGGATCTACATG
RTT13-PBS10 Lo Lav	cgcgCATGTAGATCCACTGcCCTCCGG
RTT13-PBS13 Up Lav	gtgcCCGGAGGgCAGTGGATCTACATGAGT
RTT13-PBS13 Lo Lav	cgcgACTCATGTAGATCCACTGcCCTCCGG
RTT13-PBS16 Up Lav	gtgcCCGGAGGgCAGTGGATCTACATGAGTGAC
RTT13-PBS16 Lo Lav	cgcgGTCACTCATGTAGATCCACTGcCCTCCGG
RTT16-PBS10 Up Lav	gtgcTTCCCGGAGGgCAGTGGATCTACATG
RTT16-PBS10 Lo Lav	cgcgCATGTAGATCCACTGcCCTCCGGGAA
RTT16-PBS13 Up Lav	gtgcTTCCCGGAGGgCAGTGGATCTACATGAGT
RTT16-PBS13 Lo Lav	cgcgACTCATGTAGATCCACTGcCCTCCGGGAA
RTT16-PBS16 Up Lav	gtgcTTCCCGGAGGgCAGTGGATCTACATGAGTGAC
RTT16-PBS16 Lo Lav	cgcgGTCACTCATGTAGATCCACTGcCCTCCGGGAA
PE3 (Tevo):	
PE3 Up Lav	caccgCGCCGGTCACCTGAGCTTCG
PE3 Lo Lav	aaacCGCCGGTCACCTGAGCTTCGc

**COS494 (R1905X) VQR (PAM: NGAG)**	
**Nom**	**5' -> 3'**
Comp 2 (spacer):	
Comp2 Up 494	caccgCTGAGAGCAAAATCCCAGCAgtttt
Comp2 Lo 494	ctctaaaacTGCTGGGATTTTGCTCTCAGc
Comp 3 (RTT + PBS) :	
RTT10-PBS10 Up 494A	gtgcCCACaCGTGCTGGGATTTTG
RTT10-PBS10 Lo 494A	cgcgCAAAATCCCAGCACGtGTGG
RTT10-PBS13 Up 494A	gtgcCCACaCGTGCTGGGATTTTGCTC
RTT10-PBS13 Lo 494A	cgcgGAGCAAAATCCCAGCACGtGTGG
RTT10-PBS16 Up 494A	gtgcCCACaCGTGCTGGGATTTTGCTCTCA
RTT10-PBS16 Lo 494A	cgcgTGAGAGCAAAATCCCAGCACGtGTGG
RTT13-PBS10 Up 494A	gtgcACACCACaCGTGCTGGGATTTTG
RTT13-PBS10 Lo 494A	cgcgCAAAATCCCAGCACGtGTGGTGT
RTT13-PBS13 Up 494A	gtgcACACCACaCGTGCTGGGATTTTGCTC
RTT13-PBS13 Lo 494A	cgcgGAGCAAAATCCCAGCACGtGTGGTGT
RTT13-PBS16 Up 494A	gtgcACACCACaCGTGCTGGGATTTTGCTCTCA
RTT13-PBS16 Lo 494A	cgcgTGAGAGCAAAATCCCAGCACGtGTGGTGT
RTT16-PBS10 Up 494A	gtgcGGAACACCACaCGTGCTGGGATTTTG
RTT16-PBS10 Lo 494A	cgcgCAAAATCCCAGCACGtGTGGTGTTCC
RTT16-PBS13 Up 494A	gtgcGGAACACCACaCGTGCTGGGATTTTGCTC
RTT16-PBS13 Lo 494A	cgcgGAGCAAAATCCCAGCACGtGTGGTGTTCC
RTT16-PBS16 Up 494A	gtgcGGAACACCACaCGTGCTGGGATTTTGCTCTCA
RTT16-PBS16 Lo 494A	cgcgTGAGAGCAAAATCCCAGCACGtGTGGTGTTCC
PE3 (Tevo):	
PE3 Up 494	caccgTTACCCAGAAAATCATCAAA
PE3 Lo 494	aaacTTTGATGATTTTCTGGGTAAc

**COS648 (E1833X)PAM NGG (déjà commandé, ne pas commander)**	
**Nom**	**5' -> 3'**
Comp 2 (spacer):	
Comp2 Up 648	caccGTGGATGACCTGAGCCTCACGgtttt
Comp2 Lo 648	ctctaaaacCGTGAGGCTCAGGTCATCCAC
Comp 3 (RTT + PBS) :	
RTT10-PBS10 Up 648G	gtgcTCTCGCCCGTGAGGCTCAGG
RTT10-PBS10 Lo 648G	cgcgCCTGAGCCTCACGGGCGAGA
RTT10-PBS13 Up 648G	GTGCTCTCGCCCGTGAGGCTCAGGTCA
RTT10-PBS13 Lo 648G	CGCGTGACCTGAGCCTCACGGGCGAGA
RTT10-PBS16 Up 648G	GTGCTCTCGCCCGTGAGGCTCAGGTCATCC
RTT10-PBS16 Lo 648G	CGCGGGATGACCTGAGCCTCACGGGCGAGA
RTT13-PBS10 Up 648G	GTGCTCTTCTCGCCCGTGAGGCTCAGG
RTT13-PBS10 Lo 648G	CGCGCCTGAGCCTCACGGGCGAGAAGA
RTT13-PBS13 Up 648G	GTGCTCTTCTCGCCCGTGAGGCTCAGGTCA
RTT13-PBS13 Lo 648G	CGCGTGACCTGAGCCTCACGGGCGAGAAGA
RTT13-PBS16 Up 648G	GTGCTCTTCTCGCCCGTGAGGCTCAGGTCATCC
RTT13-PBS16 Lo 648G	CGCGGGATGACCTGAGCCTCACGGGCGAGAAGA
RTT16-PBS10 Up 648G	GTGCTCATCTTCTCGCCCGTGAGGCTCAGG
RTT16-PBS10 Lo 648G	CGCGCCTGAGCCTCACGGGCGAGAAGATGA
RTT16-PBS13 Up 648G	GTGCTCATCTTCTCGCCCGTGAGGCTCAGGTCA
RTT16-PBS13 Lo 648G	CGCGTGACCTGAGCCTCACGGGCGAGAAGATGA
RTT16-PBS16 Up 648G	GTGCTCATCTTCTCGCCCGTGAGGCTCAGGTCATCC
RTT16-PBS16 Lo 648G	CGCGGGATGACCTGAGCCTCACGGGCGAGAAGATGA
Comp 4 (SpCas9 scaffold - Tevo):	
PE3 Up 648	caccGAAGTTGCCTTCACCTCCCA
PE3 Lo 648	aaacTGGGAGGTGAAGGCAACTTC

Supplementary Table S2: Compound heterozygous patient mutations.

Cell line	Mutation1	Mutation2
R1905X	c.5713C>T; p.Arg1905X	c.1481-1G>A
E1833X	c.5946+1G>A	c.5497G>T; p.Glu1833X
